# Physiological aging around the World

**DOI:** 10.1371/journal.pone.0268276

**Published:** 2022-06-08

**Authors:** Carl-Johan Dalgaard, Casper Worm Hansen, Holger Strulik

**Affiliations:** 1 Department of Economics, University of Copenhagen, København, Denmark; 2 Department of Economics, University of Göttingen, Göttingen, Germany; National University of Sciences and Technology, PAKISTAN

## Abstract

We extract data on physiological aging by computing a frailty index for 201 countries over the period 1990–2019. Using panel estimation techniques, we show that the macro frailty index replicates basic regularities previously observed in related studies of aging at the individual level. We then use the frailty index to highlight trends of global physiological aging and its relationship to economic growth. Holding population age structure fixed, the global frailty index has on average increased by about 2 percent over the last 30 years. The average person has therefore aged by what corresponds to about one life-year of physiological aging. This overall trend is relatively similar across different geographical regions. We also document a negative relationship between physiological aging of the workforce and economic growth. According to our preferred specification, a one percent increase in the frailty index of the workforce is associated with a 1.5 percent decline of GDP per capita. This means that average annual growth of labor productivity would have been 0.1 percentage points higher without physiological aging in the period 1990-2019.

## Introduction

As fertility declines and progressively more people reach an advanced age in life, the average world citizen grows older. The median age of the world population has risen persistently since the 1970s, and this process is expected to continue throughout the 21st century. Between 1990 and 2020, the median age has risen from 24 to 31 years and is projected to rise to 42 years in 2100, according to the United Nations medium fertility forecast [1]. In 2100, there will be as many people above the age of 40 as below; in the richest parts of the planet this is already the case today. Naturally, these trajectories entail changes in the demographic structure of the world population, but they also suggest physiological changes in the “average person” alive at a given point in time.

In order to assess the physiological aging of the world, we use the so-called frailty index following the seminal work of Mitnitski, Rockwood and coauthors [2–8]. As humans age, they develop an increasing number of disorders, referred to as deficits. Some of these deficits may be viewed as relatively mild nuisances while others are more serious in nature. Nevertheless, the notion is that when the number of deficits rises the body becomes more frail. For an individual, the index simply records the fraction of a set of health conditions that he or she has. As the index rises towards one the individual is viewed as increasingly frail, and in this sense physiologically older. The index itself contains information on conditions for which disease prevalence generally rises with age; communicable diseases, for example, are thus not admissible. The quality of the frailty index has been demonstrated by its predictive power for death at the individual level and for mortality at the group level, as well as for other adverse health outcomes such as the risk of institutionalization in nursing homes and becoming a disability insurance recipient [8–10]. Another reason for the popularity of the frailty index is that it can be easily compared across samples, datasets, and populations [11].

An important regularity that has emerged in the literature is that the frailty index for a representative individual grows from one birthday to the next at an approximately constant rate of 2 to 4 percent from the age 20 onwards [2, 3, 6, 12, 13]. Another regularity is that women, at given age, display more health deficits than men [4, 6, 14–16] and that men develop new health deficits faster than women [2, 3, 12, 17]. For men and women, it has been shown that there exists a strong association between the frailty index and the mortality rate, which is linear in logs such that mortality can be conceptualized as a power-law function of the frailty index [3]. The estimated gender specific parameters support the morbidity-mortality paradox, i.e. that women, on average and at given age, are more frail but face a lower risk of death [2–4, 6, 15, 18].

While, until recently, computations of the frailty index were applied exclusively to individuals, it has now been suggested to extend the methodology and compute the index for countries, continents, and at the world level [19, 20]. Such a frailty index can be constructed with data from the Global Burden of Disease (GBD) study [21], which provides for more than 200 countries prevalence rates for 369 diseases collected at five year intervals from 1990 until the present. Using the GBD 2017 data, the study by O’Donovan et al. [20] provided some basic statistics of the frailty index (including its positive association with age) for the year 2017 and focused on the validation of the index by estimating its predictive power for mortality and by comparing its composition with a battery of frailty indices computed at the individual level in previous studies. Although comorbidities were found to be over-represented in the GDB fraility index (compared to measures of function and activity), the study observed that in simple statistics (such as gender and age-group specific frailty scores) the frailty index of countries is consistent with frailty indices computed at the individual level.

Here, we report results using an updated version of the GBD data from 2019. We expand the literature in various directions. The use of a panel data set allowed us to control for country-, period-, and age-fixed effects. With country fixed effects we controlled for endowments and initial values and with period fixed effects we controlled for time trends. This way, panel regressions are conducive to achieving unbiased estimates (in contrast to cross-country regressions as in [20]). Moreover, the interpretation of results changes. For example, controlling for country fixed effect we obtained the association between age and frailty or between frailty and mortality within countries. We also computed the evolution of the frailty index for age-groups with a special focus on the working age. This allowed us to compute the frailty index of an average worker and the physiological aging of the workforce.

Controlling for country- time-, and age-fixed effects, we validate the constructed frailty index of nations against a number of regularities of the index that were previously found at the level of individuals. We then proceed by documenting global changes in physiological aging. Specifically, we report how life cycle health deficits evolve from 1990 to 2019 for the average person and for the workforce at the global level as well as for selected groups of countries. Finally, we report evidence for a negative relationship between physiological aging of the workforce and changes in income (as measured by GDP per worker), whereby we disentangle the effects of physiological aging (health deficits) from those of chronological aging (work experience) on productivity. Unlike the “correlates of growth” literature (e.g. [22]), we are not concerned with predicting growth in general or studying a list of good predictors of growth but with identifying the role of physiological and chronological aging for growth. Our approach follows previous studies on population aging and income growth (e.g. [23, 24]).

## Data and methods

### Introduction

For individuals, a frailty index is calculated as the proportion of the total potential deficits *d* = 1, …, *N* that an individual has. That is, the frailty index of individual *i* from country *c* is:

$$\begin{array}{c} d_{ic}=\frac{1}{N}\sum_{d=1}^{N}1_{ic}(d), \end{array}$$ (1)

where **1**_*ic*_(*d*) is an indicator function that takes on the value 1 if individual *i* suffers from deficit *d*.

In order to operationalize the frailty index, one needs to choose a set of deficits to include. The literature has outlined 5 criteria for inclusion [11]: (i) The deficit needs to be associated with health status. (ii) A deficit’s prevalence must generally increase with age, although some clearly age-related adverse conditions can decrease in prevalence at very advanced ages due to survivor effects. (iii) The chosen deficits must not saturate too early. For example, as humans age, it becomes harder to focus on close objects (presbyopia); by around age 55 the disease is nearly universal and thus less than ideal to include. (iv) The deficits that make up a frailty index must cover a range of systems. If the index becomes too narrowly focused, say on cognitive deficits, it potentially no longer captures overall aging but simply cognitive aging. (v) If a single frailty index is to be used serially on the same people, the items that make up the index need to be the same from one iteration to the next. No specific deficit is required to enter into the index, since results appear to be unaffected as long as a sufficient number of deficits—30 to 40—are included [11].

### Physiological aging at the country level

The average frailty index in country *c*, *d*_*c*_, is computed as:

$$\begin{array}{c} d_{c}=\frac{1}{P_{c}}\sum_{i}^{P_{c}}d_{ic}, \end{array}$$

where *P*_*c*_ is the size of the population in country *c*. In light of Eq (1) a simple rearrangement of the sum allows us to write the average frailty index as:

$$\begin{array}{c} d_{c}=\frac{1}{N}\sum_{d=1}^{N}\frac{P_{dc}}{P_{c}}, \end{array}$$

where *P*_*dc*_ is the number of people in country *c* that suffer from deficit *d*. Accordingly, in order to work out the aggregate frailty index we simply computed the average of *N* prevalence rates, *P*_*dc*_/*P*_*c*_, in each country. The frailty index for an age group *a* in country *c* is computed as

$$\begin{array}{c} d_{ac}=\frac{1}{N}\sum_{d=1}^{N}\frac{P_{dac}}{P_{ac}}, \end{array}$$

where *P*_*dac*_/*P*_*ac*_ is the prevalence rate of *d* within age group *a* in country *c*. Frailty indices for men and women were constructed analogously. Accordingly, period life-cycle deficits are defined as the unweighted average of frailty indices across age groups from 20 to 94, while the frailty index for the average hypothetical person in the population (ages 20–94) is the weighted average of the frailty indices across age groups, where the weights are the relative size of the different age groups. The distinction between unweighted and weighted frailty index allows us to consider and compare “aging of the average worker or person” (an individual characteristic) and “aging of the workforce” (a population characteristic).

### Data

Our data on prevalence rates was obtained from the newest Global Burden of Disease Study in 2019 (GBD 2019) [21] for the period 1990 to 2019. The prevalence rates are available for men and women separately, as well as for five year age-groups. When computing the frailty index, we focused on the population above 20. In order to provide a relevant validation check of our data, we created average frailty indices for both men and women separately, albeit we resorted to the overall average in the analysis of economic growth. Naturally, in order to construct deficit indices, we only included conditions that abide by the criteria listed above. We used the same conditions as in [20], except for the risk factors (e.g., “Low physical activity”), because, as far as we can see, these are not available as prevalence rates in GBD 2019. This left us with 32 conditions entering into the frailty index for which there exists data on prevalence at the global level (listed in Appendix A in S1 Appendix).

First, we studied the frailty index across four dimensions: country, year, gender, and age. Here we also exploited age-specific mortality rates (by gender) as one validation check of our country frailty index. While GBD 2019 also provides mortality data, we use the Human Mortality Database (HMD) to obtain age-specific mortality rates for a selected group of countries and years [25]. We opted for this strategy as some of the prevalence data are possibly mechanically linked to the GBD mortality data; see [21]. Summary statistics are provided in Table A.1 (Appendix B) in S1 Appendix.

Second, we investigated the development of the frailty index at the country-year level, which then collapses the gender and age dimensions and its relation to income growth. Our macro income data are from Penn World Tables [26]. We used Real GDP at constant 2017 national prices (in 2017US$) divided by the number of people in the working ages 20 to 64 as our measure of income per worker. We started at age 20 to align with the construction of the frailty index. The number of people by five-year age groups were obtained from [1]. The remaining variables were obtained from the World Development Indicators [27]. Summary statistics are provided in Table A.2 (Appendix B) in S1 Appendix.

### Validation strategies and estimation

In this and the next subsection we report results for regressions using the frailty index of nations in types of regressions that we previously conducted using the frailty index of individuals. By comparing the sign and size of the estimated coefficients with the previous studies we aim to establish validity of the frailty index of nations. We first regressed the log frailty index on a linear age variable:

$$\begin{array}{c} \text{ln}\;d_{act}^{g}=\mu_{g}{\text{age}}_{ct}^{g}+\theta_{c}+\theta_{t}+\varepsilon_{act}^{g}, \end{array}$$ (2)

where $\text{ln}\;d_{act}^{g}$ is log deficits in age group *a* (20–24, ‥, 90–94) in country *c* at period *t* (year 1990, 1995, ‥, 2019). We control for country and period fixed effects as indicated by *θ*_*c*_ and *θ*_*t*_, respectively. The variable ${\text{age}}_{ct}^{g}$ is linear in age and the estimated coefficient *μ*_*g*_ quantifies the approximate growth rate of deficits (in age) for gender *g*. Since we used five-year age groups, dividing *μ*_*g*_ by 5 provides the approximate growth rate of deficits by age at annuals levels. The error term is $\varepsilon_{act}^{g}$. We estimated *μ*_*g*_ for each gender (female, male) in separate samples, which is why the variables are indexed with superscript *g*.

In the second validation check, we estimated the association between frailty and mortality using the following log-log relationship:

$$\begin{array}{c} \text{ln}\;m_{act}^{g}=\beta_{g}\text{ln}\;d_{act}^{g}+\lambda_{c}+\lambda_{t}+\lambda_{a}+\epsilon_{act}^{g}, \end{array}$$ (3)

where $\text{ln}\;m_{act}^{g}$ is the age-specific log mortality rate. We control for the fixed effects as given by the λs for country- (*c*), period (*t*), and age- (*a*) fixed effects. We use the term “age fixed effects” as shorthand for age-group fixed effects where the age-groups are constructed over five years (as 20–24, 25–29,…, 90–94). It is worthwhile noticing that we estimated *β*_*g*_ even conditional on age fixed effects, λ_*a*_. This allows us to asses the impact of frailty on mortality for given age, i.e. controlling for age, thereby distinguishing between the role played by physiological age (deficits) and chronological age in affecting mortality. The remaining variables are defined as above and we estimated *β*_*g*_ for each gender.

After the validation checks, we studied physiological aging over time. In particular, to examine the development of the frailty index over time, we fitted a simple regression equation, regressing log deficits on a full set of period fixed effects (*ϕ*_*t*_) and an error term *ξ*_*actg*_:

$$\begin{array}{c} \text{ln}\;d_{actg}=\phi_{t}+\xi_{actg}, \end{array}$$ (4)

including both genders in the same regression (for brevity). We omitted 1990 as the comparison period, and so the estimated *ϕ*’s provided the percent increase in period “life-cycle” deficits from 1990 to the period in question, where the life-cycle is defined from age groups included in the regression. We considered all ages 20–24 to 90–94, working ages 20–24 to 60–64, and the two specific age groups 50–54 and 60–64, respectively. Comparisons of the results were used to assess the development of physiological aging holding the age composition constant (i.e. constant down to the five-year age group level). We refer to these results as “aging of the average person” (i.e. the average worker if we consider ages 20–64).

Since we were also interested in studying possible implications of physiological aging for economic growth at the country level, the next step of our analysis collapsed the deficit data to the country-period level, using data for both genders. We then calculated the weighted average of deficits from all age groups in working age (20–24,.., 60–64), where the weights were determined by the age composition. If more people are growing older in a country, the index assigns more weight to the older age groups and older age groups always have more deficits. We refer to these results as “aging of the workforce”.

Finally, we explored the relationship between the frailty index and income (GDP per worker) by estimating the following fixed-effect panel model:

$$\begin{array}{c} \text{ln}\;y_{ct}=\gamma \text{ln}\;d_{ct}+X_{ct}^{\prime }\Gamma +\sigma_{c}+\sigma_{t}+\zeta_{ct}, \end{array}$$ (5)

where ln *y*_*ct*_ is log GDP per working-age population, ln *d*_*ct*_ is the logged frailty index calculated for the average worker, and $X_{ct}^{\prime }$ contains controls for the age composition and income convergence effects. We estimate *γ* by fixed-effect panel estimation for the years 1990, 2000, 2010, and 2019, which allow us to control for country and year fixed effects. We obtain similar conclusions when estimating *γ* based on long differences from 1990 to 2019.

## Results

### Period life-cycle deficit

**Fig 1:**
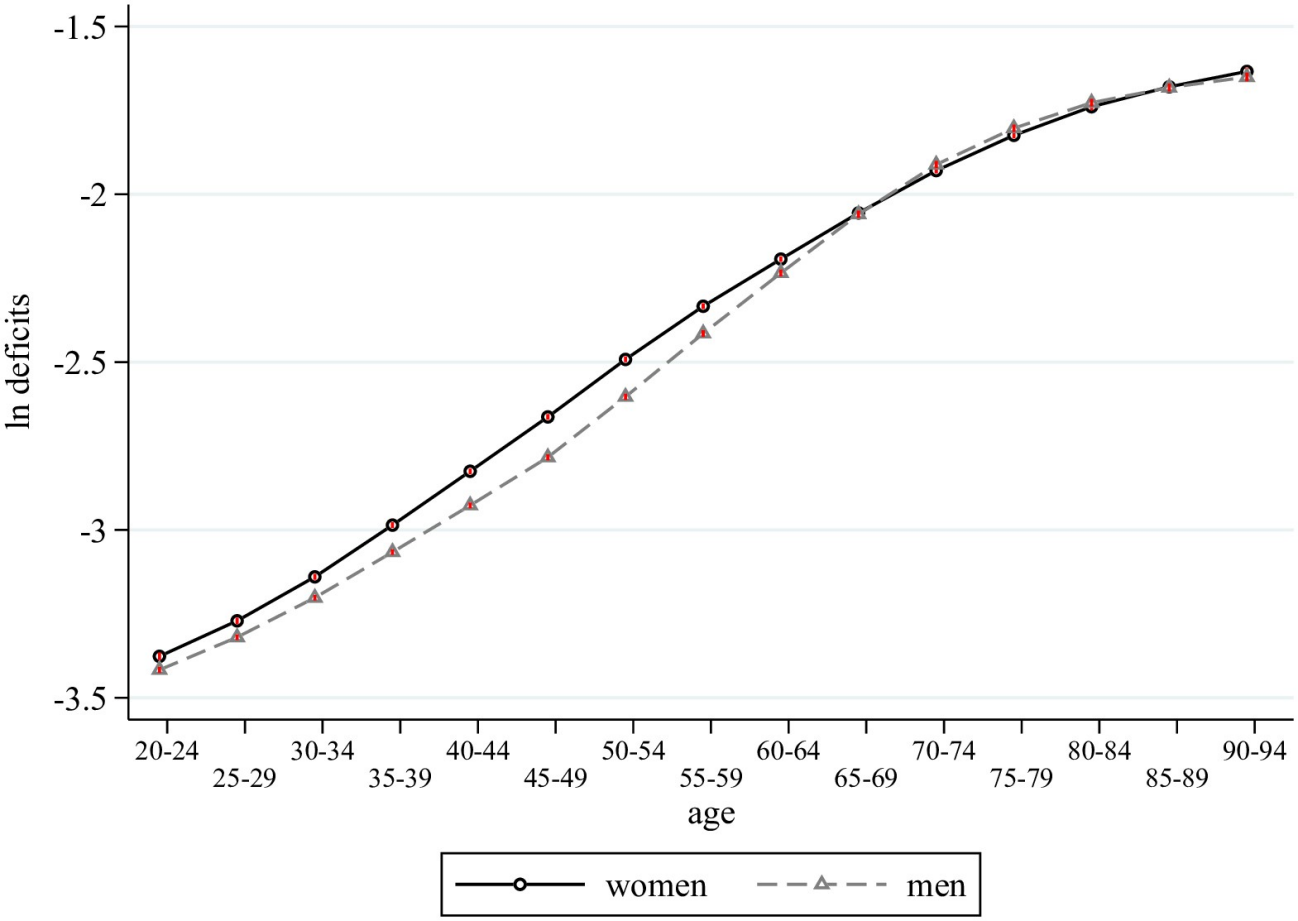
Average period life cycle deficits of women and men, 1990–2019. Notes: This figure plots the estimates from regressing log deficits on a full set of age-group dummies (omitting the regression constant) by gender, using data for all countries in the world and all periods (1990, 1995, ‥ 2019), which corresponds to the average of log deficits for each age across countries and period. The small red vertical lines indicate 95 percent confidence bands.

We begin with a graphical analysis where we plot the coefficients from regressing log deficits on a full set of age dummies, using all our available data, but estimated separately for each gender. The pattern in the estimated coefficients is shown in Fig 1. It provides visual evidence of the period life-cycle evolution of health deficits. We see the well-known regularities that growth in deficits is faster for men than women and that the intercept is larger for women, which together imply convergence of deficits over the life cycle of men and women.

Table 1 shows results of estimating Eq (2) separately for men and women. Panel A (B) reports estimates for females (males). In the first column, we consider the full sample, which includes 201 countries. For women, deficits grow by 13.4 percent from one five-year age group to the next, that is at an annual growth rate of around 13.4/5 = 2.7 percent. The corresponding number for men is 14.1/5 = 2.8 percent, and so consistent with the evidence in Fig 1 the growth rate in deficits is higher for men than women. Therefore, the behavior of our macro frailty index is squaring well findings for individual aging at the country level [4, 12, 28, 29]. Moving from left to right in the table, the countries included in sample change. Columns 2–4 focus on geographical areas (continents), whereas the final three column consider OECD countries, high-income (high and upper-middle) countries, and low-income (low and lower-middle) countries as currently defined by the World Bank [27]. The key result is that the growth rate of deficits across the life course is remarkably stable: it varies between 2.6 to 2.9 percent per year of age in all parts of the world.

**Table 1:** Estimated growth rate of deficits.

Panel A: Female Sample								
	(1)	(2)	(3)	(4)	(5)	(6)	(7)	(8)
Age	0.134***	0.133***	0.137***	0.136***	0.131***	0.136***	0.136***	0.132***
	(0.000)	(0.001)	(0.001)	(0.001)	(0.001)	(0.001)	(0.001)	(0.001)
Constant	-3.484***	-3.411***	-3.514***	-3.491***	-3.496***	-3.451***	-3.474***	-3.499***
	(0.004)	(0.007)	(0.007)	(0.007)	(0.007)	(0.008)	(0.005)	(0.005)
Observations	21,105	4,305	6,405	4,095	5,565	3,150	12,600	8,295
*R* ^2^	0.981	0.988	0.982	0.979	0.976	0.991	0.984	0.976
Sample	World	Europe	Asia	Americas	Africa	OECD	Rich	Poor
Panel B: Male Sample								
	(1)	(2)	(3)	(4)	(5)	(6)	(7)	(8)
Age	0.141***	0.144***	0.144***	0.145***	0.132***	0.146***	0.144***	0.136***
	(0.001)	(0.001)	(0.001)	(0.001)	(0.001)	(0.001)	(0.000)	(0.001)
Constant	-3.581***	-3.544***	-3.619***	-3.616***	-3.540***	-3.578***	-3.588***	-3.571***
	(0.004)	(0.005)	(0.005)	(0.007)	(0.008)	(0.009)	(0.004)	(0.007)
Observations	21,105	4,305	6,405	4,095	5,565	3,150	12,600	8,295
*R* ^2^	0.979	0.981	0.981	0.979	0.980	0.983	0.981	0.978
Sample	World	Europe	Asia	Americas	Africa	OECD	Rich	Poor

Notes: This table reports the results from estimating *μ*_*g*_ in Eq 2, where the dependent variable is age specific log deficits and the explanatory variable is a linear age variable taking a separate value for each age group (ages 20–24 to 90–94). All regressions include country and period fixed effects. The countries included in the sample are indicated at the bottom: World is all countries (Column 1); Europe is European countries (Column 2), etc. Standard errors, clustered at the country level, are reported in parenthesis.

### The frailty index and mortality

**Fig 2:**
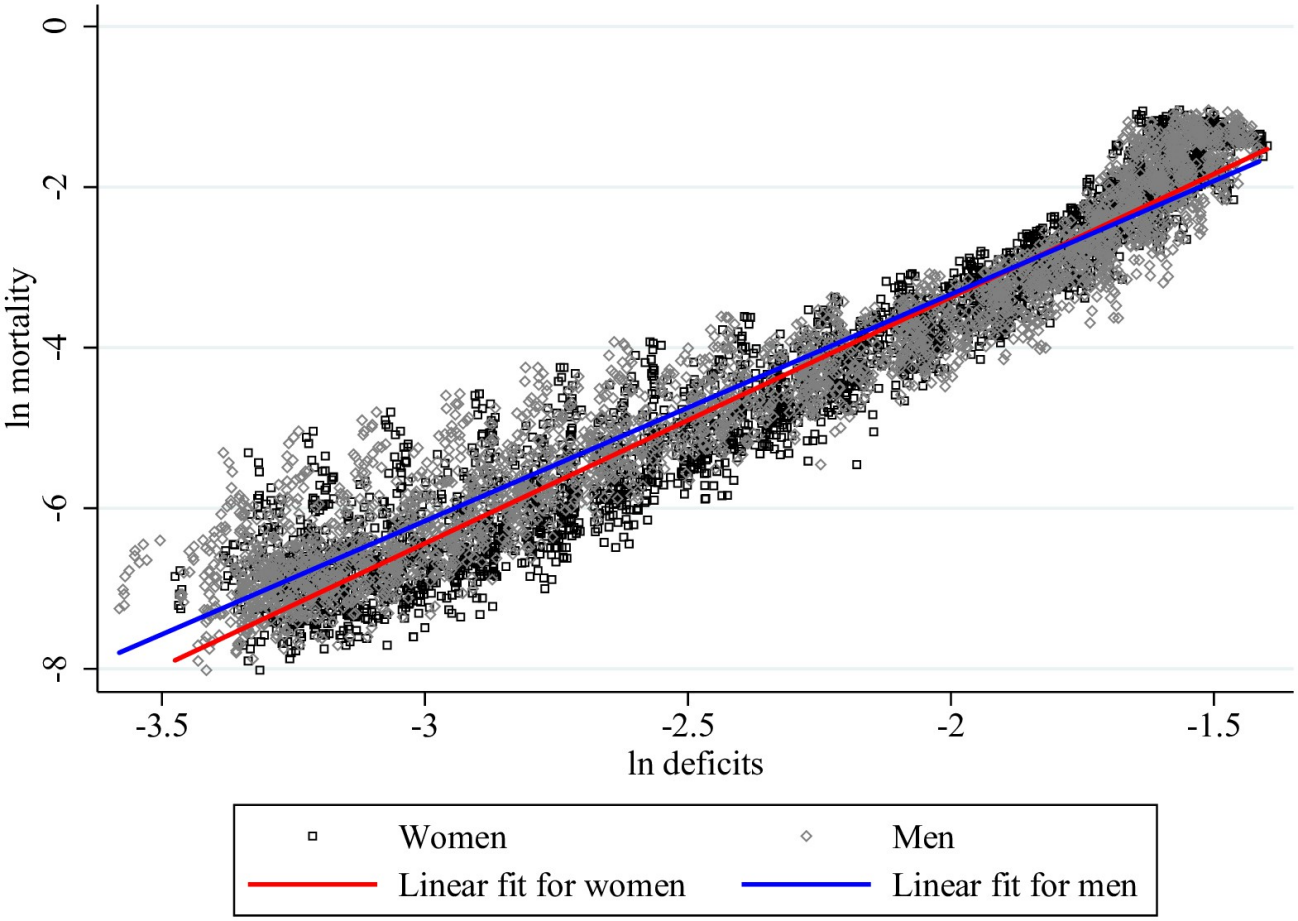
Frailty and mortality of women and men. Notes: This figure plots mortality-deficits observations (both logged) by gender for selected group of countries where age-specific mortality rates are available at the Human Mortality Database. The fitted lines are simple OLS regression lines.

Results from estimating Eq (3) are reported in Table 2, again separately for men and women. The first column shows the unconditional relationship, which is also illustrated as a scatterplot in Fig 2. The regression implies a power law association between mortality and the frailty index, *m* ∝ *d*^*β*^ with *β* = 3.1 for women and 2.8 for men. The power law is a natural consequence when mortality increases at a constant rate with age (according to the Gompertz law, [30]) and the frailty index increases at constant rate, as in model ((2)).

**Table 2:** Estimated frailty-mortality relationship.

Panel A: Female Sample				
	(1)	(2)	(3)	(4)
Deficits	3.067***	3.067***	3.065***	1.551***
	(0.044)	(0.046)	(0.046)	(0.475)
Constant	2.762***	2.763***	2.759***	-0.801
	(0.076)	(0.109)	(0.109)	(1.117)
Observations	3,105	3,105	3,105	3,105
*R* ^2^	0.929	0.961	0.968	0.986
Country FE	NO	YES	YES	YES
Period FE	NO	NO	YES	YES
Age FE	NO	NO	NO	YES
Panel B: Male Sample				
	(1)	(2)	(3)	(4)
Deficits	2.823***	2.817***	2.816***	0.999**
	(0.046)	(0.049)	(0.049)	(0.455)
Constant	2.310***	2.296***	2.292***	-2.056*
	(0.089)	(0.118)	(0.118)	(1.088)
Observations	3,105	3,105	3,105	3,105
*R* ^2^	0.931	0.955	0.962	0.986
Country FE	NO	YES	YES	YES
Period FE	NO	NO	YES	YES
Age FE	NO	NO	NO	YES

Notes: This table reports the results from estimating *β*_*s*_ in Model 3, where the dependent variable is the age-specific logged mortality rates and the explanatory variable is age specific logged deficits. The sample is a selected group of countries where mortality rates are available in the Human Mortality Database. Standard errors, clustered at the country level, are reported in parenthesis.

Mortality is on average lower but increases more steeply in the frailty index for women. Together with the observation that frailty is on average higher for women, we observe the morbidity-mortality paradox, which applies until the two regression lines intersect in Fig 2 (at a mortality rate of about 0.14). The log-log association allows us to interpret the coefficients as elasticities. When the frailty index increases by one percent, the mortality rate of women increases on average by 3.1 percent and that of men by 2.8 percent. The estimated coefficient sizes are unaffected by controlling for country and period fixed effects (Columns 2 and 4). In the final column, we show that the estimated positive relationship is robust to controlling for age fixed effect, although the magnitude reduces substantially. The estimated coefficient for deficits provides the deficit-mortality elasticity at given age (within countries). It allows us to assess the independent contribution of the frailty index to mortality when age is controlled for. This exercise is important since both mortality and frailty depend positively on age. The point estimate for ln deficits of women (men) in column (4) is about half (35 percent) of that in column (3). It suggests that about half (35 percent) of the frailty-mortality nexus is explained when controlling for age.

### Physiological aging over time

**Fig 3:**
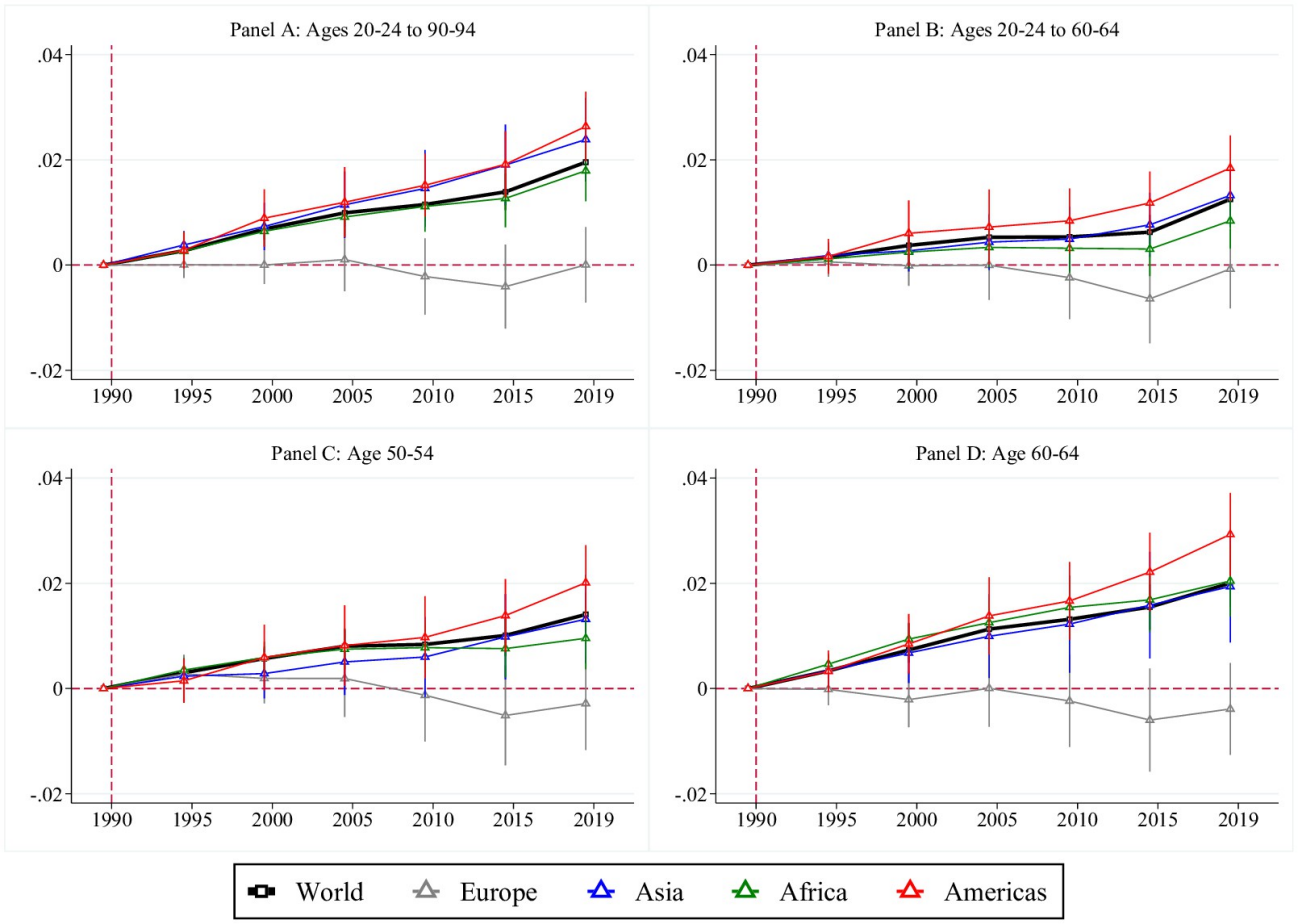
Physiological aging around the world. Notes: This figure plots the estimates from regressing logged deficits on period dummies (i.e., 1990, 1995, 2000, 2005, 2010, 2015, 2019), where 1990 is the omitted comparison period for all samples, along with their 95% confidence bands. Panel A includes all ages from 20–24 to 90–94. Panel B includes all working ages from 20–24 to 60–64. Panel C includes the age 50–54. Panel D includes the age 60–64.

Having demonstrated the validity of the frailty index based on data from all countries in the world, we next report results on global trends in physiological aging from 1990 to 2019. For this, we regressed log deficits on period dummies (or fixed effects) and omitted the initial period, year 1990. These estimates reveal the change in physiological aging in percent, compared to the base year. Results are depicted in Fig 3. In Panel A, we include all five-year age group (20–24 to 90–94), and so the estimates reveal the change in period life-cycle deficits; that is, the number of deficits a hypothetical person would suffer if she was subjected to the prevailing age-group-specific deficits throughout her life. From 1990 to 2019, we find an average increase of about 2 percent when including all countries in the world, which is driven mostly by countries in the Americas and Asia, while physiological aging in Europe has not changed. Thus, the average person became physiologically older by 2 percent, compared to 1990. In Fig A.1 in S1 Appendix, we report the deficits by the level of income, according the World Bank’s [27] definition, as above, and by OECD membership. While rich and poor countries developed similarly with an increase of health deficits by about 2 percent, the average citizen of OECD countries did not age, in physiological terms, between 1990 and 2019.

Panel B reveals similar patterns when only considering the working ages from 20–24 to 60–64. Deficits of workers increased by about 1.2 percent from 1990 to 2019. In Panels C and D, we show the changes in the deficits for the age-groups 50–54 and 60–64, respectively. Again, the patterns are similar, but with even more heterogeneity across continents and income groups. In particular, elderly persons of working age (60–64) in the OECD countries became more healthy and physiologically younger by almost 2 percent, compared to 1990 (Fig A.3 in S1 Appendix, panel D).

### Physiological aging of the work force and economic growth

We next present the implications of our frailty index for physiological aging around the world over the last quarter of a century. We focus on the evolution of the average frailty index for the economically active part of the population, i.e. the population between the age of 20 and 64. The age groups 20–24 to 60–64 approximate the working age population. The frailty index of the workforce is a weighted average of deficits for the ages 20–24 to 60–64, where the weights are the population shares of the different age groups. Therefore, the workforce ages along two dimensions: First, within age group, the average worker ages, as documented in the previous subsection. Second, across age-groups, the share of elderly workers increases.

**Fig 4:**
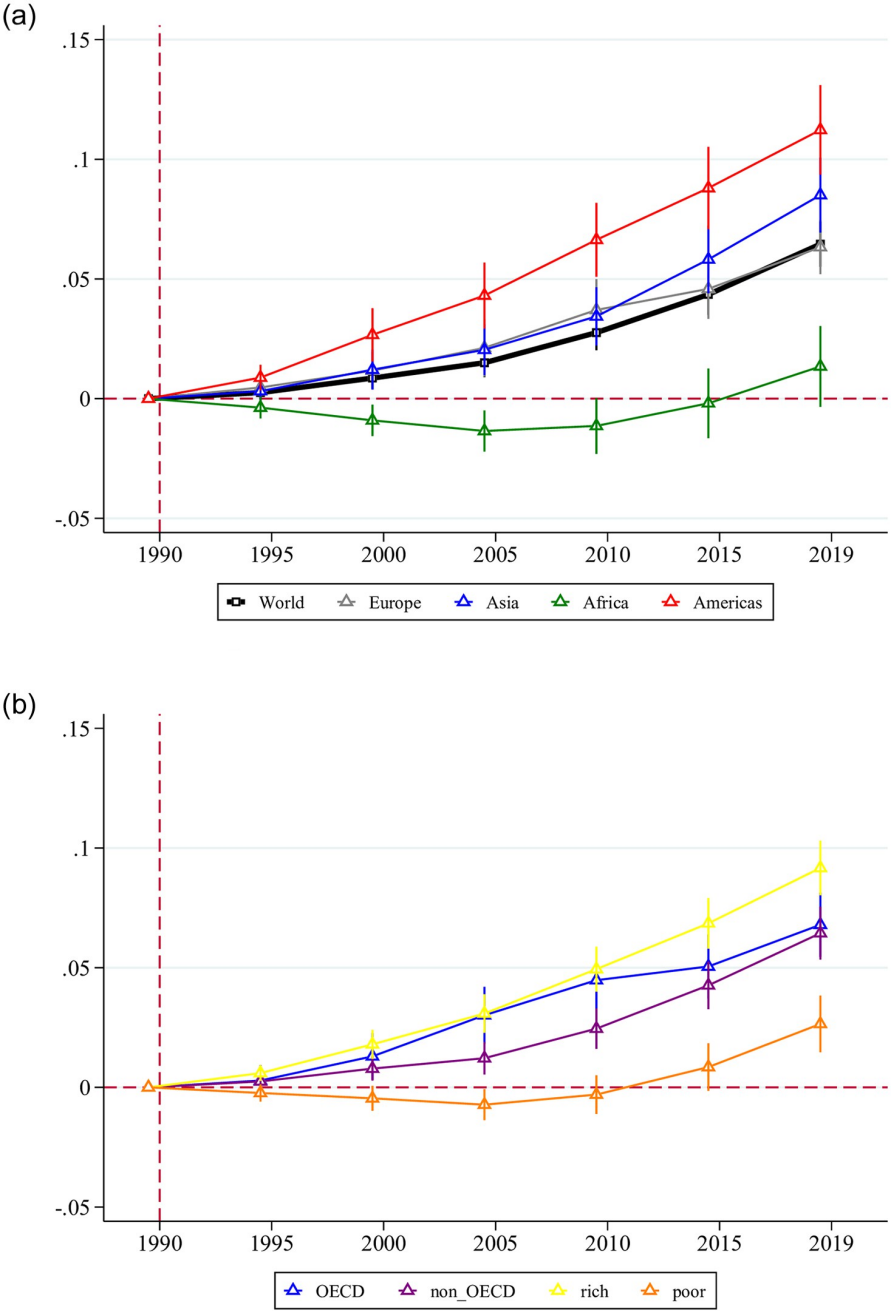
Physiological aging of the workforce. Notes: This figure plots the estimates from regressing logged deficits of the average worker on period dummies (i.e., 1990, 1995, 2000, 2005, 2010, 2015, 2019), where 1990 is the omitted period of comparison for all samples, along with their 95% confidence bands. Left: sample split by continent. Right: sample split by level of economic development.

Fig 4 plots on the left-hand side the change in physiological aging of the workforce of the world by regions. We observe the largest increase in physiological aging for countries in the Americas. We also observe that the (mild) physiological aging of the average worker in African countries has been neutralized by a compositional shift towards a greater share of younger workers in the workforce. The panel on the right-hand side of Fig 4 shows results for the income split of countries. We observe that health deficits of the workforce increased across all income categories of countries and at most in the rich countries where it was about 9 percent higher in 2019 than in 1990.

Table 3 reports the results of estimating *γ* in Eq 5. In the first column, we report the association between the frailty index and GDP per working-age population without any controls besides country and period fixed effects, which are controlled for in all specifications. We find a positive, but statistically insignificant estimate. In the second column, we include controls for convergence by including initial GDP per worker (measured in 1990) interacted with period fixed effects. It is important to control for this variation since the initial level of deficits is correlated with the subsequent change in deficits (due to the compensating law of deficits) and the initial level of GDP per worker, which is correlated with subsequent per worker GDP growth due to convergence effects [31]. Now, the correlation becomes statistically significant but it remains positive. As already documented extensively, deficits are strongly positively correlated with age and thus column 3 includes controls for the age structure of the population. We consider this as our benchmark specification. The relationship between frailty index and growth now becomes negative and statistically significant. The estimated elasticity implies that an increase in deficits of one percent is associated with a decrease of GDP per working-age population by 1.5 percent. However, results from regression work such as this are sensitive to data quality for GDP (e.g. [32]). As a robustness check, we report results when Africa is excluded from the sample. Then, the productivity effect of physiological aging becomes larger and more precisely estimated, as shown in column 4 of Table 3.

**Table 3:** Physiological aging and economic growth.

	(1)	(2)	(3)	(4)
ln Deficits	0.99	1.79***	-1.53**	-2.20***
	(0.61)	(0.66)	(0.73)	(0.75)
pop. share 25–34			2.39**	1.57
			(1.15)	(1.16)
pop. share 35–44			1.05	0.55
			(0.77)	(0.86)
pop. share 45–54			3.79***	3.32***
			(1.13)	(1.22)
pop. share 55–64			7.36***	7.66***
			(1.31)	(1.31)
Observations	672	672	672	472
Country FE	Yes	Yes	Yes	Yes
Period FE	Yes	Yes	Yes	Yes
Initial GDP/worker x Period FE	No	Yes	Yes	Yes
Excl. Africa	No	No	No	Yes

Notes: This table reports the results from estimating *γ* in Model 5, where the dependent variable is GDP per worker, using panel-model estimation for the periods (or years) 1990, 2000, 2010, and 2019. All regressions include country and period fixed effects. “pop. share 20–24” is the omitted reference group for the age-share controls. Standard errors, clustered at the country level, are reported in parenthesis.

In the Appendix we report additional robustness checks. First, we applied a long difference approach which includes only the earliest and latest periods (1990 and 2019). The point estimates of the aging coefficient (ln Deficits) are larger in absolute value but they do not significantly differ from the benchmark estimates, see columns (3) and (4), Appendix Table A.3. Second, we control for cross-country differences in physical capital, life expectancy, population size, and years of schooling. The initial values of these confounding variables in 1990 were interacted with full set of period fixed effects. We follow this approach (instead of controlling for the variables directly) in order to minimize the issue of including “bad controls” in the regression because these variables can be seen as outcome variables themselves (see [33]). The estimates of the aging coefficient were found to differ insignificantly from that of our benchmark regression, corroborating the robustness of our main results, see Table A.4 in S1 Appendix.

## Discussion

We constructed a frailty index for countries based on disease prevalence rates and showed that the aggregate (or macro-) index preserves key regularities that have previously been observed at the micro level in samples of individuals. In keeping with the results from previous studies at the micro level [2, 3, 6, 12], the growth rate of the frailty index is very stable. For all subdivision of the world’s countries, it increases at a constant rate between 2.6 to 2.9 percent per additional year of age at all ages. The differentiated evolution of deficits over the life course for the two genders observed at the individual level [2, 3, 12, 17] was observable at the macro level as well: the growth rate of deficits was found to be faster for men than women, but the intercept was found to be larger for women. These two stylized facts confirm jointly the compensation effect of morbidity, in analogy to the Strehler-Mildvan correlation of mortality [2, 12, 34].

Combining the frailty index with mortality data from the Human Mortality Database, we found frailty and mortality to be linearly associated in logs, again replicating at the macro level previous findings at the micro level [3]. The power law suggests that a one percent increase in the frailty index is associated with an increase of the mortality rate by about 3 percent for women and 2.8 percent for men. These changes are also in terms of magnitude similar to previous findings at the micro level [3]. Since mortality has been found to be on average, for given age, higher for women, the results corroborated, at the macro level, the morbidity–mortality paradox [2–4, 6, 15, 18].

A distinctive feature of our analysis is the use of panel data estimation techniques. Controlling subsequently for country- and period-fixed-effects we were thus able to establish the robustness of the power law between frailty and mortality, which holds (with insignificant change in the estimated parameters) across countries, within countries, and within countries for different periods of time. Significantly, we introduced age fixed effects into the mortality regressions. While this procedure, naturally, reduced the estimated coefficient of log deficits substantially, it also strengthens the case in favor of an independent effect of health deficits on mortality. The results suggest that, for *given* age, mortality of women (men) increases by 1.5 percent (1.0 percent) when health deficits increase by 1 percent.

We then explored long-run trends of physiological aging in a period life-cycle way, holding the age composition constant. Period life-cycle deficits thus measure the deficits of a hypothetical cohort subjected to the age-specific deficits prevailing at a given period of time. We found that, in the average country, the frailty index increased by 2 percent since 1990. This corresponds with a little less than one life-year of physiological aging since our data suggests deficits increase by about 2.5 percent per year.

The trends in period life-cycle deficits have implications for the physiological aging of the workforce, which takes into account not only trends in individual aging but also trends in the age composition of the workforce. Here, we found that health deficits of the workforce of the world increased by about 6.5 percent (or circa 2 life-years of physiological aging) from 1990 to 2019. In the Americas, the increase is more than 10 percent because of fast physiological aging at both margins: faster aging of the average worker and a greater share of elderly workers in the workforce. In Africa, in contrast, faster aging at the individual level is compensated by a compositional shift toward younger workers in the workforce. The same observation can be alternatively stated in more negative terms. Deteriorating health at the individual level prevents that the African countries benefit from the demographic dividend in the same way as countries that underwent the demographic transition earlier [35].

In order to correctly interpret the results, it is useful to remember that during the period of investigation 1990–2019, the workforce aged by 7 years in chronologically terms (see Introduction) while it aged by two years in physiological terms. We thus find evidence of healthy aging [36].

For Europe, the healthy aging effects are even stronger. We found that the physiological age of the European population did not change from 1990 to 2019 while the median (chronological) age increased from 34.6 to 42.5 during the observation period [1]. Our macro results may still appear low compared to trends observed at the micro level suggesting that individuals from 14 European countries displayed 1.4–1.5 percent fewer health deficits per later year of birth [37]. When we reduced the sample to the 14 European countries considered in [37], we found that period life-cycle deficits decreased by about 2 percent since 1990. The direction of the trend thus goes in the same direction, albeit it seems to operate at a slower pace at the macro level. One reason for the different speed of trends could be the different composition of the frailty index. Whereas the macro frailty index consists solely of diseases, the micro index consists of diseases as well as functional limitations. It should also be noted that our study focussed on period effects and not cohort effects as in [37].

It is has been frequently argued that population aging is a key factor in shaping the current and future development of societies, often through its first order impact on labor markets [38]. Macroeconomic studies based on estimated impacts of chronological aging on productivity and labor force participation usually arrive at grim projections for economic growth due to population aging. For example, Maestas et al. predict that annual GDP growth will slow by 1.2 percentage points this decade and 0.6 percentage points next decade due to population aging [39], see also [40, 41]. Here, we contributed to the aging–growth debate by estimating the association between the frailty index and labor productivity of the workforce.

We found a positive link between chronological age of the workforce (measured by the share of workers between 55 and 64) and labor productivity, and a negative link between physiological age (frailty) and labor productivity. These findings shed new light on the age–productivity nexus by disentangling chronological age and physiological age (health). In economics, individual productivity is typically estimated via the Mincer-wage equation [42], i.e. it is postulated that log-wages increase linearly with chronological age and decline linearly with chronological age squared. The wage measures productivity and the age terms are interpreted as experience at work [42, 43]. With rising age, the squared term eventually becomes dominating and causes productivity to decline. While it is plausible that the linear, positive effect of age captures increasing productivity through experience, it is hard to understand why productivity should decline because of “too much experience”. The decline of productivity with advancing chronological age is more convincingly rationalized as a proxy of something else that causes productivity to decline as workers grow older, namely declining health [44, 45]. Here, we showed that this is indeed the case. Controlling for health deficits, the greatest positive contribution to labor productivity comes from the chronologically oldest, i.e. the most experienced workers.

Our findings suggest that physiological aging exerted a mildly negative impact on economic growth during the period of observation. Controlling for age, health deficits of the average worker increased by about 2 percent from 1990 to 2019 and combining this result with our baseline growth-estimate from column (3) in Table 3 indicates that physiological aging is associated with a reduction in GDP per working-age population of 2 × 1.5 = 3 percent. Since the average economy in the world grew by about 40 percent from 1990 to 2019, i.e. by about 1.14 percent per year, this means that average annual growth would have been 0.1 percentage points higher without physiological aging. This productivity effect appears to be rather mild in light of recent macroeconomic studies on the effect of population aging on economic growth. Most importantly, all regression results highlight that physiological aging and not chronological aging is a drag on growth. Being chronologically old means being more experienced and thus a greater share of elderly workers is conducive to productivity growth when health is controlled for in the regressions.

## Supporting information

S1 Appendix (PDF)
