# Supplementary material for: Physiological aging around the World
Source: PLoS One. 2022 Jun 8;17(6):e0268276. doi: 10.1371/journal.pone.0268276 (PMC9176773; doi:10.1371/journal.pone.0268276)
Supplement: S1 Appendix — (PDF) [file pone.0268276.s001.pdf]

Physiological Aging Around the World

Appendix

Carl-Johan Dalgaard[1]
Casper Worm Hansen[2]
Holger Strulik[3]

[1] Department of Economics, University of Copenhagen, Øster Farimagsgade 5, 1353 København, Denmark.
[2] Department of Economics, University of Copenhagen, Øster Farimagsgade 5, 1353 København, Denmark.
[3] University of Göttingen, Department of Economics, Platz der Göttinger Sieben 3, 37073 Göttingen, Germany; Correspondence to holger.strulik@wiwi.uni-goettingen.de.

A. Items in the Frailty Index

The frailty index is based on prevalence rates for the following diseases (32 in total):

Diarrheal diseases; Protein-energy malnutrition; Neoplasms; Ischemic heart disease; Stroke; Non-rheumatic valvular heart disease; Cardiomyopathy and myocarditis; Atrial fibrillation and flutter; Peripheral artery disease; Other cardiovascular and circulatory diseases; Chronic respiratory diseases; Peptic ulcer disease; Gallbladder and biliary diseases; Alzheimer's disease and other dementias; Parkinson's disease; Depressive disorders; Diabetes mellitus; Chronic kidney disease; Skin and subcutaneous diseases; Other sense organ diseases; Rheumatoid arthritis; Osteoarthritis; Low back pain; Gout; Urinary diseases and male infertility; Genital prolapse; Endocrine, metabolic, blood, and immune disorders; Oral disorders; Falls; Hearing loss; Heart failure; Blindness and vision loss.

B. Additional results

Table A.1. Summary Statistics I

Panel A: Female

VARIABLES	(1) N	(2) mean	(3) max	(4) min
Ln deficits	21,105	-2.409	-1.390	-3.534
Ln mortality	3,105	-4.447	-1.041	-8.016

Panel B: Male

VARIABLES	(1) N	(2) mean	(3) max	(4) min
Ln deficits	21,105	-2.453	-1.394	-3.581
Ln mortality	3,105	-4.447	-1.041	-8.016

Notes: This table reports summary statistics for the data set used in Sections 3.1-3.3. The sample dimensions are: 1990, 1995, .. 2019 for the age-groups 20-24 to 90-94 in 201 countries for the deficit variable; and 1990, 1995, 2015 for the age groups 20-24 to 90-94 in 37 countries for the mortality variable.

Figure A.1. Physiological Aging by the level of economic development

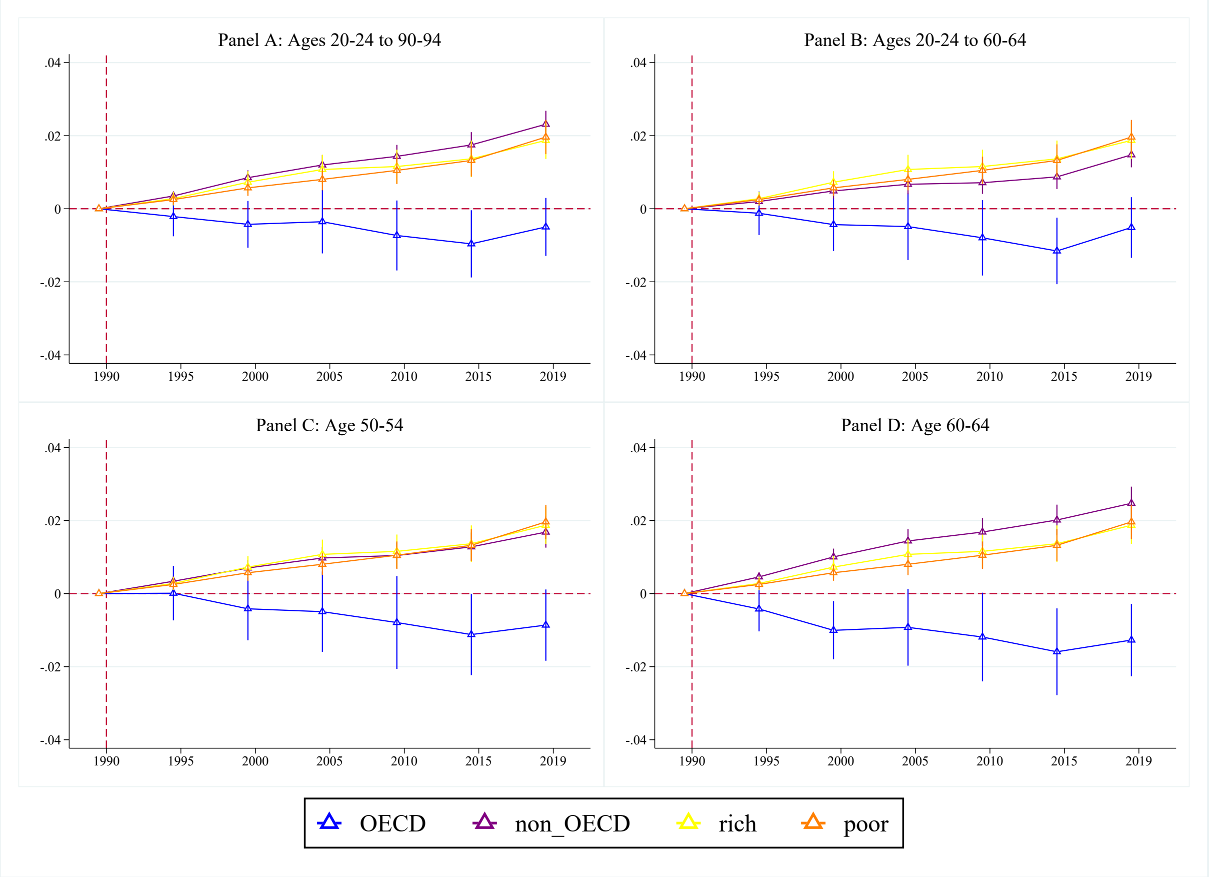

Notes: This figure plots the estimates from regressing logged deficits on period dummies (i.e., 1990, 1995, 2000, 2005, 2010, 2015, 2019), where 1990 is the omitted comparison period for all samples, along with their 95% confidence bands. Panel A includes all ages from 20-24 to 90-94. Panel B includes all working ages from 20-24 to 60-64. Panel C includes the age 50-54. Panel D includes the age 60-64.

Table A.2: Summary Statistics II

VARIABLES	(1) N	(2) mean	(3) max	(4) min
initial ln GDP/worker	672	2.658	5.133	0.0480
pop. share 20-24	672	0.166	0.270	0.0632
pop. share 25-34	672	0.294	0.449	0.175
pop. share 35-44	672	0.233	0.349	0.163
pop. share 45-54	672	0.176	0.273	0.105
pop. share 55-64	672	0.130	0.261	0.0250
ln deficits	672	-2.901	-2.599	-3.094
ln GDP/worker	672	2.840	5.133	-0.870

Notes: This table reports summary statistics for the data set used in the GDP regressions for the periods 1990, 2000, 2010, 2019. Data availability for GDP/worker restricts the sample to 168 countries.

Table A.3. Physiological Aging and Economic Growth: Long differences (1990 – 2019)

	(1)	(2)	(3)	(4)
dln Deficits	1.00	1.92**	-2.13**	-3.32***
	(0.73)	(0.82)	(1.07)	(1.11)
dpop. share 25-34			4.20	0.06
			(2.57)	(2.62)
dpop. share 35-44			-0.33	-2.43
			(1.57)	(1.90)
dpop. share 45-54			6.44***	4.69*
			(2.31)	(2.79)
dpop. share 55-64			9.44***	6.84**
			(2.73)	(2.94)
Observations	168	168	168	118
Initial GDP/worker	No	Yes	Yes	Yes
Excl. Africa	No	No	No	Yes

Notes: This table reports the results from estimating γ in Model **??**, using long differences (1990 and 2019) to fix out country specific effects. Consequently, the dependent variable is the change in logged GDP per worker, the main explanatory variable is the change in logged deficits for the average worker, and common year trend is captured by the regression constant. "dop. share 20-24" is the omitted reference group for the age-share controls. Standard errors, clustered at the country level, are reported in parenthesis.

Table A.4. Physiological Aging and Economic Growth: Robustness

	(1)	(2)	(3)	(4)	(5)
ln Deficits	-1.68**	-1.62**	-1.46*	-1.68**	-1.66**
	(0.76)	(0.76)	(0.82)	(0.79)	(0.79)
pop. share 25-34	2.17*	1.85	2.28*	1.06	1.04
	(1.21)	(1.27)	(1.30)	(0.90)	(0.88)
pop. share 35-44	1.05	0.40	0.34	-0.01	-0.03
	(0.75)	(0.79)	(0.81)	(0.77)	(0.74)
pop. share 45-54	3.66***	2.78**	3.07**	2.66**	2.53**
	(1.09)	(1.17)	(1.22)	(1.11)	(1.09)
pop. share 55-64	7.46***	6.42***	6.39***	5.40***	5.39***
	(1.23)	(1.19)	(1.27)	(1.23)	(1.22)
Observations	668	660	660	536	536
Initial GDP/worker	Yes	Yes	Yes	Yes	Yes
Initial capital/worker	Yes	Yes	Yes	Yes	Yes
Initial life expectancy	No	Yes	Yes	Yes	Yes
Initial population size	No	No	Yes	Yes	Yes
Initial schooling	No	No	No	Yes	Yes

Notes: This table reports the results from estimating γ in Model **??**, using panel-model estimation for the periods (or years) 1990, 2000, 2010, and 2019. All regressions include country and period fixed effects. "pop. share 55-64" is the omitted reference group in the age-share controls. All the initial controls (GDP/worker, capital/worker, life expectancy, population size, schooling) are measured in 1990, logged and interacted with a full set of year fixed effects. GDP/worker is defined in the main text. Capital per worker is the capital stock at constant 2017 national prices (in mil. 2017US$) divided by the size of working population. Life expectancy in years is measured at birth. Schooling is average years of schooling in the workforce. Standard errors, clustered at the country level, are reported in parenthesis. Data are obtained from Feenstra et al. (2015), Worldbank (2021), and Barro and Lee (2013).

Table A.5. Aggregate Deficits by Country and Period

country	1990	2000	2010	2019
Albania	0.0547	0.0578	0.0615	0.0643
Algeria	0.0473	0.0474	0.0494	0.0536
Angola	0.0504	0.0491	0.0485	0.0491
Antigua and Barbuda	0.0509	0.0524	0.0562	0.0602
Argentina	0.0548	0.0541	0.0552	0.0560
Armenia	0.0560	0.0570	0.0570	0.0610
Australia	0.0586	0.0575	0.0589	0.0603
Austria	0.0650	0.0665	0.0679	0.0682
Azerbaijan	0.0525	0.0519	0.0532	0.0586
Bahamas	0.0506	0.0539	0.0560	0.0592
Bahrain	0.0459	0.0479	0.0487	0.0514
Bangladesh	0.0499	0.0491	0.0494	0.0525
Barbados	0.0515	0.0557	0.0601	0.0629
Belarus	0.0675	0.0673	0.0686	0.0711
Belgium	0.0614	0.0625	0.0669	0.0662
Belize	0.0483	0.0486	0.0514	0.0531
Benin	0.0514	0.0512	0.0512	0.0516
Bhutan	0.0506	0.0504	0.0497	0.0518
Bolivia	0.0532	0.0531	0.0535	0.0545
Bosnia and Herzegovina	0.0600	0.0621	0.0648	0.0698
Botswana	0.0548	0.0554	0.0561	0.0582
Brazil	0.0516	0.0546	0.0542	0.0561
Brunei Darussalam	0.0514	0.0526	0.0561	0.0617
Bulgaria	0.0667	0.0650	0.0672	0.0696
Burkina Faso	0.0499	0.0494	0.0490	0.0500
Burundi	0.0491	0.0489	0.0469	0.0474
Cabo Verde	0.0522	0.0514	0.0516	0.0550
Cambodia	0.0484	0.0487	0.0484	0.0503
Cameroon	0.0519	0.0507	0.0500	0.0511
Canada	0.0502	0.0520	0.0548	0.0566
Central African Republic	0.0510	0.0499	0.0493	0.0494
Chad	0.0523	0.0515	0.0511	0.0505
Chile	0.0529	0.0585	0.0567	0.0612
China	0.0501	0.0504	0.0537	0.0588
Colombia	0.0519	0.0539	0.0585	0.0606
Comoros	0.0503	0.0494	0.0489	0.0503
Congo	0.0498	0.0485	0.0489	0.0510
Costa Rica	0.0530	0.0551	0.0573	0.0606
Cote d'Ivoire	0.0516	0.0517	0.0514	0.0514
Croatia	0.0677	0.0693	0.0725	0.0742
Cyprus	0.0537	0.0550	0.0563	0.0583
Czechia	0.0662	0.0664	0.0700	0.0719

Table A.5 (cont.): Aggregate Deficits by Country and Period

country	1990	2000	2010	2019
DR Congo	0.0493	0.0490	0.0487	0.0488
Denmark	0.0626	0.0644	0.0666	0.0660
Djibouti	0.0491	0.0489	0.0496	0.0520
Dominican Republic	0.0484	0.0495	0.0518	0.0545
Ecuador	0.0502	0.0522	0.0547	0.0570
Egypt	0.0476	0.0474	0.0484	0.0507
El Salvador	0.0537	0.0546	0.0558	0.0570
Equatorial Guinea	0.0520	0.0500	0.0479	0.0473
Estonia	0.0672	0.0670	0.0664	0.0688
Eswatini	0.0567	0.0577	0.0568	0.0564
Ethiopia	0.0508	0.0495	0.0484	0.0480
Fiji	0.0507	0.0540	0.0567	0.0594
Finland	0.0612	0.0648	0.0676	0.0654
France	0.0605	0.0604	0.0632	0.0637
Gabon	0.0539	0.0512	0.0493	0.0508
Gambia	0.0520	0.0535	0.0517	0.0520
Georgia	0.0594	0.0592	0.0622	0.0662
Germany	0.0619	0.0655	0.0664	0.0680
Ghana	0.0463	0.0469	0.0481	0.0500
Greece	0.0594	0.0581	0.0597	0.0630
Grenada	0.0542	0.0550	0.0572	0.0598
Guatemala	0.0537	0.0526	0.0525	0.0534
Guinea	0.0523	0.0518	0.0512	0.0508
Guinea-Bissau	0.0530	0.0527	0.0521	0.0521
Guyana	0.0510	0.0536	0.0573	0.0597
Haiti	0.0527	0.0525	0.0517	0.0535
Honduras	0.0509	0.0505	0.0516	0.0537
Hungary	0.0666	0.0656	0.0674	0.0684
Iceland	0.0579	0.0607	0.0626	0.0630
India	0.0547	0.0554	0.0557	0.0583
Indonesia	0.0519	0.0519	0.0532	0.0568
Iran (Islamic Republic of)	0.0507	0.0489	0.0501	0.0551
Iraq	0.0494	0.0477	0.0483	0.0496
Ireland	0.0552	0.0560	0.0571	0.0598
Israel	0.0563	0.0570	0.0590	0.0594
Italy	0.0638	0.0630	0.0633	0.0677
Jamaica	0.0507	0.0523	0.0548	0.0572
Japan	0.0638	0.0641	0.0657	0.0658
Jordan	0.0467	0.0466	0.0473	0.0499
Kazakhstan	0.0553	0.0558	0.0565	0.0600
Kenya	0.0470	0.0456	0.0460	0.0476
Kuwait	0.0461	0.0497	0.0492	0.0586
Kyrgyzstan	0.0544	0.0519	0.0520	0.0546

Table A.5 (cont.): Aggregate Deficits by Country and Period

country	1990	2000	2010	2019
Laos	0.0472	0.0461	0.0463	0.0482
Latvia	0.0698	0.0702	0.0675	0.0721
Lebanon	0.0501	0.0487	0.0532	0.0558
Lesotho	0.0569	0.0556	0.0553	0.0574
Liberia	0.0519	0.0514	0.0512	0.0520
Lithuania	0.0694	0.0712	0.0715	0.0743
Luxembourg	0.0605	0.0622	0.0625	0.0629
Madagascar	0.0535	0.0523	0.0521	0.0526
Malawi	0.0507	0.0503	0.0488	0.0491
Malaysia	0.0469	0.0485	0.0494	0.0515
Maldives	0.0495	0.0482	0.0457	0.0462
Mali	0.0529	0.0513	0.0507	0.0515
Malta	0.0574	0.0586	0.0622	0.0621
Mauritania	0.0518	0.0514	0.0512	0.0524
Mauritius	0.0495	0.0519	0.0580	0.0598
Mexico	0.0525	0.0531	0.0577	0.0603
Mongolia	0.0504	0.0494	0.0511	0.0557
Montenegro	0.0620	0.0635	0.0658	0.0680
Morocco	0.0494	0.0501	0.0531	0.0570
Mozambique	0.0544	0.0509	0.0502	0.0500
Myanmar	0.0467	0.0468	0.0488	0.0515
Namibia	0.0551	0.0532	0.0538	0.0552
Nepal	0.0531	0.0533	0.0543	0.0549
Netherlands	0.0553	0.0571	0.0591	0.0615
New Zealand	0.0596	0.0609	0.0616	0.0630
Nicaragua	0.0506	0.0502	0.0518	0.0553
Niger	0.0531	0.0548	0.0541	0.0535
Nigeria	0.0524	0.0528	0.0525	0.0527
Norway	0.0643	0.0660	0.0689	0.0695
Oman	0.0468	0.0465	0.0457	0.0482
Pakistan	0.0518	0.0511	0.0509	0.0521
Panama	0.0520	0.0533	0.0558	0.0589
Paraguay	0.0486	0.0496	0.0503	0.0516
Peru	0.0514	0.0521	0.0539	0.0570
Philippines	0.0484	0.0491	0.0518	0.0531
Poland	0.0623	0.0605	0.0608	0.0627
Portugal	0.0581	0.0571	0.0595	0.0605
Qatar	0.0490	0.0535	0.0500	0.0523
Republic of Korea	0.0543	0.0569	0.0605	0.0642
Republic of Moldova	0.0636	0.0626	0.0629	0.0658
Romania	0.0687	0.0670	0.0690	0.0708
Russian Federation	0.0671	0.0674	0.0674	0.0701

Table A.5 (cont.): Aggregate Deficits by Country and Period

country	1990	2000	2010	2019
Rwanda	0.0510	0.0501	0.0490	0.0503
Saint Lucia	0.0535	0.0540	0.0575	0.0604
Saint Vincent and the Grenadines	0.0523	0.0536	0.0581	0.0620
Sao Tome and Principe	0.0532	0.0510	0.0515	0.0538
Saudi Arabia	0.0476	0.0506	0.0538	0.0588
Senegal	0.0536	0.0526	0.0519	0.0530
Serbia	0.0652	0.0656	0.0726	0.0697
Seychelles	0.0481	0.0484	0.0514	0.0574
Sierra Leone	0.0522	0.0493	0.0481	0.0484
Singapore	0.0548	0.0590	0.0617	0.0639
Slovakia	0.0662	0.0663	0.0691	0.0711
Slovenia	0.0658	0.0662	0.0689	0.0716
South Africa	0.0573	0.0577	0.0569	0.0595
Spain	0.0550	0.0536	0.0550	0.0601
Sri Lanka	0.0500	0.0525	0.0556	0.0586
Sudan	0.0472	0.0475	0.0488	0.0497
Suriname	0.0506	0.0518	0.0547	0.0580
Sweden	0.0602	0.0615	0.0617	0.0619
Switzerland	0.0641	0.0666	0.0664	0.0656
Syrian Arab Republic	0.0469	0.0461	0.0475	0.0511
Taiwan (Province of China)	0.0492	0.0509	0.0543	0.0569
Tajikistan	0.0511	0.0489	0.0490	0.0523
Thailand	0.0495	0.0520	0.0552	0.0593
Togo	0.0514	0.0507	0.0506	0.0519
Trinidad and Tobago	0.0528	0.0564	0.0586	0.0626
Tunisia	0.0484	0.0488	0.0512	0.0559
Turkey	0.0530	0.0536	0.0572	0.0577
Turkmenistan	0.0504	0.0498	0.0514	0.0548
Uganda	0.0470	0.0465	0.0458	0.0461
Ukraine	0.0695	0.0691	0.0659	0.0691
United Arab Emirates	0.0466	0.0461	0.0462	0.0501
United Kingdom	0.0610	0.0630	0.0632	0.0659
United Republic of Tanzania	0.0521	0.0513	0.0510	0.0521
United States of America	0.0577	0.0560	0.0590	0.0620
Uruguay	0.0574	0.0560	0.0576	0.0591
Uzbekistan	0.0513	0.0513	0.0521	0.0554
Venezuela	0.0511	0.0523	0.0544	0.0591
Viet Nam	0.0459	0.0453	0.0478	0.0522
Yemen	0.0472	0.0480	0.0468	0.0475
Zambia	0.0470	0.0459	0.0454	0.0466
Zimbabwe	0.0504	0.0502	0.0503	0.0534

References Appendix

Barro, Robert J., and Lee, J. W. (2013). A new data set of educational attainment in the world, 1950–2010. *Journal of Development Economics*, 104, 184-198.

World Bank, World Development Indicators. Available at https://databank.worldbank.org/source/world-development-indicators (downloaded on [January, 2021]).

Feenstra, R.C., Inklaar, R. and Timmer, M.P. (2015). The next generation of the Penn World Table. *American Economic Review*, 105(10), 3150-3182. Available at https://www.rug.nl/ggdc/productivity/pwt/ (data downloaded on [September, 2021]).
